# Papanicolaou Society of Cytopathology new guidelines have a greater ability of risk stratification for pancreatic endoscopic ultrasound-guided fine-needle aspiration specimens

**DOI:** 10.18632/oncotarget.14105

**Published:** 2016-12-22

**Authors:** Bo Chen, Yu Zhao, Jiangang Gu, Huanwen Wu, Zhiyong Liang, Zhilan Meng

**Affiliations:** ^1^ Department of Pathology, Peking Union Medical College Hospital, Chinese Academy of Medical Sciences and Peking Union Medical College, Wangfujing, Beijing, 100730, China

**Keywords:** endoscopic ultrasound-guided fine-needle aspiration, Papanicolaou Society of Cytopathology, risk of malignancy, risk stratification, pancreas

## Abstract

The Papanicolaou Society of Cytopathology has recently proposed a standardized terminology and nomenclature guidelines for pancreatic cytology. However the risk of malignancy associated with the new guidelines has been scarcely studied. In this study, a series of pancreatic cytology cases obtained by endoscopic ultrasound-guided fine-needle aspiration from 294 Chinese patients were retrospectively re-categorized into six categories according the new guidelines. The risks of malignancy were 18.1% for “negative,” 20.0% for “neoplastic,” 57.1% for “nondiagnostic,” 69.2% for “atypical,” 87.5% for “suspicious,” and 100.0% for “positive” respectively. The area under the receiver operating characteristic curve was 0.93 (95% Confidence Interval, 0.90-0.96), which was significantly higher than that associated with old classification system (0.82; 95% Confidence Interval, 0.77-0.87) conventionally used in China. Our investigation demonstrated that the new guidelines have a greater ability of risk stratification than the old classification system conventionally used in China. This may be helpful in giving better predictions of malignancy, thus leading to more personalized treatment strategies.

## INTRODUCTION

Pancreatic cancer is one of the most virulent tumors with a five-year survival rate of 7%. [1] It is the sixth leading cause of cancer-related death in Chinese male population and seventh in female. [2] Due to the nonspecific early symptoms, by the time of diagnosis most of the pancreatic cancers are surgically unresectable. [3] Therefore early detection and accurate pathologic evaluation play a pivotal role in improving prognosis especially for patients with resectable localized pancreatic lesion.

Endoscopic ultrasound-guided fine-needle aspiration (EUS-FNA) is a relatively safe and accurate technique to acquire pancreatic cytology specimen for rapid pathologic diagnosis. Numerous studies have revealed the advantages of EUS-FNA technique for sampling pancreatic lesions, which include reasonable sensitivity, high specificity, minimal invasiveness and appreciable accuracy. [4–12] However, until recently there was no widely accepted categorization system for pancreatic cytology obtained by EUS-FNA.

In order to set uniform criteria, the Papanicolaou Society of Cytopathology (PSC) has recently developed a standardized terminology and nomenclature guidelines for pancreatobiliary cytology. [13] It includes a six-tiered system: “nondiagnostic,” “negative,” “atypical,” “neoplastic,” “suspicious,” and “positive.”

Nevertheless, to our best knowledge, few researchers [4, 14, 15] have applied the PSC new guidelines for a retrospective study of pancreatic EUS-FNA cytology, wherein only hundreds of cases were reviewed. For example, Layfield et al [14] conducted a retrospective study of 317 cases, in which they reported the risk of malignancy associated with each PSC category and confirmed the remarkable ability of risk stratification. Smith et al [15] also reported the risk of malignancy using the new classification in a study of 127 cases of pancreatic neoplastic mucinous cysts. Saieg et al [4] concluded that the application of PSC guidelines had a greater impact among specimen previously categorized as atypical and suspicious by a comparative study of 155 cases. Due to the limited number of studies and the limited number of specimens within each study, more investigations are needed to contribute to the knowledge regarding the new classification.

Furthermore, most of the Chinese cytopathologists have hitherto adopted an old classification system conventionally used in China, which includes four categories: “negative,” “atypical,” “suspicious” and “positive.” Obviously it has two drawbacks. First, the “negative” cases may include a proportion of cases with specimens inadequate to make any diagnosis. Such cases were classified into a newly defined “nondiagnostic” category by PSC new system. Secondly, the “positive” category includes high grade, aggressive malignancies as well as neoplasms with benign or borderline biological behaviors. The latter ones were categorized into a newly defined “neoplastic” category by PSC classification. Therefore it is high time to examine the advantage of the PSC new guidelines over old classification for Chinese patients, thus promoting the application of the new classification by medical practitioners in China.

In this article, the PSC new guidelines were applied to retrospectively re-categorize a series of pancreatic EUS-FNA cytology cases of 294 Chinese patients from Peking Union Medical College Hospital (PUMCH). And the risks of malignancy for each category were calculated. This study also conducted a comparative study between the PSC new guidelines and the old classification system conventionally used in China.

## RESULTS

A total of 294 patients who met the inclusion criteria were enrolled in this investigation. There were 183 men and 111 women. The median age was 55 years, ranging from 19 to 83 years.

Original cytologic diagnoses by old classification system included 104 “negative” cases, 13 “atypical” cases, 32 “suspicious” cases, and 145 “positive” cases. Upon applying PSC new guidelines, there were 21 “nondiagnostic” cases, 83 “negative” cases, 13 “atypical” cases, 20 “neoplastic” cases, 32 “suspicious” cases, and 125 “positive” cases. The correlation of the old cytologic categories with final diagnoses was demonstrated as Table 1. Then a similar correlation table was constructed for the PSC classification as Table 2. It can be observed that the old “atypical” and old “suspicious” categories were identical to their new counterparts by PSC guidelines respectively. The old “negative” was sub-stratified into the new “negative” and new “nondiagnostic,” whereas the old “positive” into the new “neoplastic” and new “positive.” The specified histologic subtypes and their frequencies within each PSC cytologic categories were listed in Supplementary Table 1 in Supplementary Data.

**Table 1:** Correlation of Cytologic Diagnoses with Final Diagnoses in 294 Patients Using Conventionally Old Categories

Cytologic Categories	Final Diagnoses (Histologic and Clinical Follow-Ups)			
	NN	N w/o HGM	N w/t HGM	Total
“Old” Negative	70	7	27	104
“Old” Atypical	3	1	9	13
“Old” Suspicious	1	3	28	32
“Old” Positive	0	16	129	145
Total	74	27	193	294

Abbreviation:NN, Non-Neoplastic; N w/o HGM, Neoplastic Without High Grade Malignancy; N w/t HGM, Neoplastic With High Grade Malignancy.

**Table 2:** Correlation of Cytologic Diagnoses with Final Diagnoses in 294 Patients Using PSC New Categories

Cytologic Categories	Final Diagnoses (Histologic and Clinical Follow-Ups)			
	NN	N w/o HGM	N w/t HGM	Total
“New” Nondiagnostic	5	4	12	21
“New” Negative	65	3	15	83
“New” Atypical	3	1	9	13
“New” Neoplastic	0	16	4	20
“New” Suspicious	1	3	28	32
“New” Positive	0	0	125	125
Total	74	27	193	294

Abbreviation:NN, Non-Neoplastic; N w/o HGM, Neoplastic Without High Grade Malignancy; N w/t HGM, Neoplastic With High Grade Malignancy.

### Risk of malignancy

Using the new guidelines, the absolute risks of malignancy were 18.1% for “negative,” 20.0% for “neoplastic,” 57.1% for “nondiagnostic,” 69.2% for “atypical,” 87.5% for “suspicious,” and 100.0% for “positive” respectively by an ascending order (see Table 3). “Nondiagnostic” (*P*=0.0001), “atypical” (*P*<0.0001), “suspicious” (*P*<0.0001) and “positive” (*P*<0.0001) categories were statistically different from “negative” category. Wherein “nondiagnostic” was also significantly different from “positive” (*P*=0.0025), but “atypical” (*P*=0.0370) and “suspicious” (*P*=0.0399) were not different from “positive.” It was noteworthy that “neoplastic” was not significantly different from “negative” (*P*=0.8408), but statistically different from “positive” (*P*=0.0002). A similar table for the old classification was also constructed as Supplementary Table 2 in Supplementary Data.

**Table 3:** Absolute Risk and Relative Risk of Malignancy for Each PSC New Categories

Cytological Category	Absolute Risk(95% CI)	Relative Risk(95% CI)	*P* (Relative to Negative)	*P* (Relative to Positive)
“New” Negative	18.1 (9.8-26.4)	1.00	-	<0.0001
“New” Neoplastic	20.0 (2.5-37.5)	1.11 (0.41-2.98)	0.8408	0.0002
“New” Nondiagnostic	57.1 (36.0-78.3)	3.16 (1.75-5.70)	0.0001	0.0025
“New” Atypical	69.2 (44.1-94.3)	3.83 (2.14-6.87)	<0.0001	0.0370
“New” Suspicious	87.5 (76.0-99.0)	4.84 (3.01-7.80)	<0.0001	0.0399
“New” Positive	100.0 (100.0-100.0)	5.40 (3.44-8.46)	<0.0001	-

### Sensitivity, specificity and ROC curve analysis

As illustrated in Table 4, for the PSC new guidelines, when the grouping of “atypical,” “suspicious” and “positive” categories was altogether treated as positive test, the sensitivity, specificity and accuracy rate associated with this combination were 83.93%, 92.08% and 86.73% respectively. This combination yielded the maximum value of the Youden index, meaning the best combination of the sensitivity and specificity. A similar table for the old classification was also constructed as Supplementary Table 3 in Supplementary Data. Likewise the best combination of the sensitivity and specificity was achieved when the grouping of “atypical,” “suspicious” and “positive” categories was altogether treated as positive test for the old classification.

**Table 4:** Sensitivity, Specificity and Accuracy Rate Associated with Various Combinations of PSC Cytologic Categories

Cut-Point	Sensitivity	Specificity	Accuracy Rate	Youden Index
≥“New” Negative	100.00%	0.00%	65.64%	0.0000
≥“New” Neoplastic	92.23%	67.33%	83.67%	0.5956
≥“New” Nondiagnostic	90.16%	83.17%	87.76%	0.7333
≥“New” Atypical	83.93%	92.08%	86.73%	0.7601
≥“New” Suspicious	79.27%	96.04%	85.03%	0.7531
≥“New” Positive	64.77%	100.00%	76.87%	0.6477

Cytologic categories were rearranged by an ascending order of absolute risk and were successively set as diagnostic threshold (cut-point).

**Figure 1:**
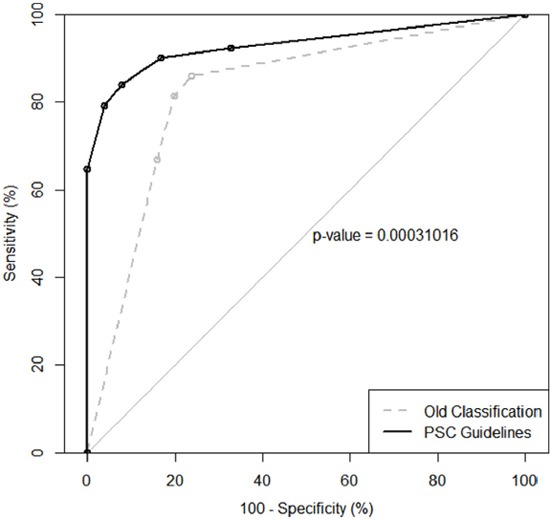
ROC curve for the PSC guidelines compared to ROC curve for the old classification PSC guidelines have significantly stronger ability of risk stratification than old classification (P=0.0003).

Figure 1 illustrated a receiver operating characteristic (ROC) curve for the PSC guidelines comparing with an ROC curve for the originally old classification. The area under the receiver operating characteristic curve (AUROC) for the old and the new classifications were 0.82 (95% Confidence Interval (CI), 0.77-0.87) and 0.93 (95% CI, 0.90-0.96) respectively; and they were statistically different (*P*=0.0003). It can therefore be confirmed that PSC guidelines have significantly stronger ability of risk stratification and provide with better diagnostic accuracy. The ROC curve analysis once again demonstrated that the best combination of sensitivity and specificity was achieved when the grouping of “atypical,” “suspicious” and “positive” was treated as positive test.

## DISCUSSION

Early detection of pancreatic cancer and accurate diagnosis of its histologic subtype are of ultimate importance on patient's management and outcome. It is noteworthy that EUS-FNA procedure has been reported by numerous researchers to have excellent sensitivity and specificity for diagnosing pancreatic cytology. Puli et al reported the EUS-FNA had a pooled sensitivity of 86.8% and a pooled specificity of 95.8% through a meta-analysis of 41 studies. [9] Consistently Chen et al reported the pooled sensitivity and specificity of EUS-FNA for solid pancreatic lesion were 92% and 96% respectively by a meta-analysis of 15 studies with 1860 patients. [8] A plenty of studies, especially earlier-published, utilized the “benign or malignant” binary system for EUS-FNA cytology report. However, it has been proved by some authors [16–18] that a stratification system would be beneficial for personalized risk analysis and clinical follow-up.

The Papanicolaou Society of Cytopathology has recently proposed a standardized terminology and nomenclature guidelines for pancreatic cytology. However, the risk of malignancy associated with each PSC category has been scarcely investigated. Layfield et al reported the risks of malignancy were 13% for “negative,” 14% for “neoplastic,” 21% for “nondiagnostic,” 74% for “atypical,” 82% for “suspicious”, and 97% for “positive” respectively. [14] Smith et al reported 0% for “negative,” 13% for “neoplastic,” 17.4% for “nondiagnostic,” 63.6% for “atypical,” 80% for “suspicious”, and 100% for “positive,” which were comparable to Layfield et al's results, although their study focused only on pancreatic neoplastic mucinous cysts. [15] In our study, we retrospectively re-classified a series of 294 pancreatic EUS-FNA cytology cases, and the risks of malignancy were 18.1% for “negative,” 20.0% for “neoplastic,” 57.1% for “nondiagnostic,” 69.2% for “atypical,” 87.5% for “suspicious,” and 100.0% for “positive,” which were in general agreement with published literatures mentioned above.

It can be suggested that the new classification did better stratify the risk of malignancy for pancreatic EUS-FNA specimens for following reasons.

First, the PSC new guidelines have an advantage over the old one by further stratifying the old “positive” category into a new “neoplastic” category with benign or borderline biological behaviors and a new “positive” category with high grade aggressive behaviors. As a result, the new “positive” category was associated with a markedly higher risk than the old “positive” (100.0% versus 89.0%), making a more discernable difference with other categories.

Secondly, the old “negative” category (26.0%) was associated with an appreciably higher risk than the new “negative” (18.1%) as well as its counterparts in other literatures (13% by Layfied et al, 0% by Smith et al [15]). It suggests that the PSC new guidelines have an advantage over the old one by further stratifying the old “negative” category into two distinctive new categories: “nondiagnostic” and “negative.” The former one had a statistically higher risk of malignancy than the latter one, thus decreasing the risk of the real “negative” cases.

Lastly, the AUROC for the new classification was 0.93 (95% CI, 0.90-0.96), a value represented a nearly perfect ability of risk stratification. And it was statistically higher than the AUROC for the old classification (0.82), which further proved the advantage of new guidelines over the old classification in the ability of risk stratification.

For new classification, when “atypical,” “suspicious” and “positive” categories were combined as positive test, the sensitivity was 83.93% and the specificity was 92.08%. It corroborated the perfect specificity of EUS-FNA technique as reported by many authors. [8, 9, 12, 19, 20] Although the sensitivity was somewhat lower than that in Layfield et al's study, [14] it was in general agreement with many other studies in a statistical sense. [9, 19–22]

This article confirmed the PSC guidelines stratified the risk of malignancy as demonstrated by Layfield et al and Smith et al. [14, 15] To our best knowledge, this study for the first time conducted a comparative study of the PSC guidelines and an old classification system conventionally used in China. Again, it was for the first time to investigate the application of PSC new guidelines on pancreatic EUS-FNA cytology for Chinese population.

However, there were several limitations in our study as described below.

First, there were no cases which can be classified into “neoplastic: benign” subcategory. In addition, mainly for the financial reasons, most of the patients histologically diagnosed as intraductal papillary mucinous neoplasm (IPMN) or mucinous cystic neoplasm (MCN) at PUMCH were not performed pre-operative EUS-FNA cytology, and thus were not enrolled in this study. Instead, they would like to choose surgical resection directly. Consequently, the number of “neoplastic: other” cases was small comparing to other literatures. Therefore it had insufficient statistical power for neoplastic cases due to the insufficient sample size.

Secondly, the number of “atypical” cases was small comparing to other studies. PUMCH is one of the most leading hospitals in China, therefore the prevalence of high grade malignant cases were higher due to Berkson's bias. It might partly account for the relatively small number of “atypical” cases and large number of “positive” cases.

Thirdly, the “nondiagnostic” category was associated with a markedly higher risk of malignancy (57.1%) than its counterparts in Layfield et al's study (21%) [14] and in Smith et al's study (17.4%). [15] It might be attributed to the retrospective nature of this study without the availability of rapid on-site evaluation (ROSE) by a cytopathologist. ROSE is reported to have significantly increased the diagnostic accuracy rate and sampling adequacy rate for pancreatic lesions, even if performed by endosonagraphers. [8, 23–27] In our study, many of “nondiagnostic” cases would have been classified into “atypical,” “suspicious” or “positive” categories, if ROSE had been applied. It is also possible that a higher prevalence of malignancy in the current study cohort, which partly due to the fact that the lower-risk IPMNs and MCNs were less likely evaluated pre-operatively by EUS-FNA in our institution, might contribute to the higher risk of “nondiagnostic” category.

Lastly, the number of specimens at a single center was always limited. Thus more studies at different centers worldwide and subsequent meta-analysis should be done to confirm the results in this article and to further contribute to the relevant knowledge.

An important aspect, not discussed within this study, is the genetic alterations in the diagnosis and categorization of pancreatic cytology. The oncogene *KRAS* point mutation was reported by many literatures to be the major molecular event in pancreatic ductal adenocarcinoma (PDAC) and cholangiocarcinoma, [28–31] as well as pancreatic cystic lesions. [32] In this study, the specificity of pancreatic EUS-FNA cytology was almost perfect, especially when using PSC new guidelines. Nevertheless, the sensitivity was still less than ideal especially when “positive” category alone was treated as positive outcome. Contrarily, *KRAS* mutation analysis was shown to increases the sensitivity, but decrease the specificity when comparing to cytology diagnoses. [31] Since that *KRAS* mutation analysis can compensate the conventional EUS-FNA cytology by improving the diagnostic accuracy, [30, 31] it should also improve the categorization accuracy for PSC new guidelines. Future investigation should be conducted to testify the value of *KRAS* mutation analysis on each category based on the PSC new classification.

In conclusion, the application of PSC new guidelines for pancreatic EUS-FNA specimens demonstrated stratified risks of malignancy associated with diagnostic cytologic categories, as well as high sensitivity and specificity associated with various combinations of these cytologic categories. The diagnostic accuracy and the ability of risk stratification of this new classification were proved to be significantly higher than the old classification conventionally used in China. Our results may be helpful in encouraging more cytopathologists to apply the PSC new guidelines, giving better predictions of malignancy, thus leading to more personalized treatment strategies.

## MATERIALS AND METHODS

### Ethics statement

This study was approved by the institutional review board of PUMCH and all aspects of the study comply with the Declaration of Helsinki. All of the enrolled patients had signed the informed consent by the time of fine needle aspiration procedure and by the time of surgical operation, which authorized scientific research on the blood, tissue and other samples obtained from their human bodies.

### Patients and sample collection

An electronic database search was performed for all the patients who underwent pancreatic EUS-FNA procedures using 22-gauge needles from September 2008 to December 2015 at PUMCH. The inclusion criteria were following: (1) the histological diagnosis was obtained by biopsy, surgery or autopsy at anytime during follow-up; (2) for patients without histological diagnosis, no clinical and radiological evidence of malignancy was documented for at least 36 months after initial EUS-FNA cytology diagnosis. As a result, a total of 294 patients were enrolled (183 men, 111 women; median age 55 years, range 19-83 years).

### Cytologic classification

All the slides were reviewed by two experienced pathologists (B.C. and Z.M.) independently, and the original diagnoses were blind to investigators when reviewing slides.

Original cytologic diagnoses had been classified by an old classification system conventionally used in China, which included four categories: “negative,” “atypical,” “suspicious,” and “positive.” For this retrospective study, all the cytologic diagnoses were re-classified according to the PSC new guidelines into 6 categories: “nondiagnostic,” “negative,” “atypical,” “neoplastic,” “suspicious,” and “positive.”

According to the PSC new guidelines, the “neoplastic” category is further separated into two subcategories: “neoplastic: benign” and “neoplastic: other.” In this study, it happened that no specimen could be categorized into “neoplastic: benign” subcategory. Therefore in this article “neoplastic” category was identical to “neoplastic: other” subcategory.

Morphologically, the “atypical” and “suspicious” categories share the almost identical categorization criteria for both old and new classifications. For the old “negative” category, the diagnostic criterion was that no evidence of any cellular atypia was found, no matter if adequate cellular sample was acquired or not. It seemed to be in good agreement with the combination of new “negative” and new “nondiagnostic” categories. As for the old “positive” category, clearly neoplastic cells were observed, despite of the degree of malignancy. It compared well with the combination of new “neoplastic” and new “positive” categories.

### Histologic and clinical follow-ups

Histologic and clinical follow-ups were defined as “final diagnoses” in this article, and were further stratified into three subgroups: “non-neoplastic,” “neoplastic without high grade malignancy” and “neoplastic with high grade malignancy.” All of the histologic diagnoses were revised according to World Health Organization (WHO) 2010 classification. [33] “Non-neoplastic” subgroup was mainly composed of normal pancreatic tissue, acute pancreatitis (AP), chronic pancreatitis (CP), autoimmune pancreatitis (AIP), pseudocyst, lymphoepithelial cyst, etc. For a patient without histologic diagnosis, as described earlier, if adequate clinical and radiological follow-up information could be obtained to exclude malignancy for at least 36 months, then his/her case was classified into “non-neoplastic” subgroup. “Neoplastic without high grade malignancy” was mainly composed of intraductal papillary mucinous neoplasm (IPMN), mucinous cystic neoplasm (MCN), pancreatic neuroendocrine tumor G1 or G2 (PanNET), and solid pseudopapillary neoplasm (SPN). “Neoplastic with high grade malignancy” subgroup generally referred to pancreatic ductal adenocarcinoma (PDAC), as well as other high-grade, aggressive malignancies, such as IPMN/MCN with invasive carcinoma, neuroendocrine carcinoma (NEC) and SPN with high grade malignant transformation, etc. In this study, “non-neoplastic” and “neoplastic without high grade malignancy” subgroups were altogether treated as negative outcome. Meanwhile, “neoplastic with high grade malignancy” subgroup was treated as positive outcome.

### Statistical analysis

Statistical analysis was carried out using R (R Foundation for Statistical Computing, Vienna, Austria) [34] and two of its packages: metafor [35] and pROC. [36] The level of significance was defined as P≤0.05, two-tailed.

Absolute risk, relative risk, and the *P* value relative to the “negative” category were calculated to determine the risk of malignancy and to test the statistical differences between “negative” category and other ones. Subsequently, absolute risk of each category was compared with “positive” category and *P* values were calculated in order to test the statistical differences between “positive” category and other ones.

The cytologic diagnostic categories, which were re-arranged by an ascending order of risk of malignancy, were successively set as diagnostic threshold, namely cut-point. Categories with risks of malignancy equal to or greater than the “cut-point” category were combined together as a grouping, which was defined as positive test. Whereas categories with risks of malignancy lower than the “cut-point” category were combined together as a grouping, which was defined as negative test. For each of these combinations, the sensitivity, specificity, accuracy rate and the Youden index were calculated.

On the basis of the sensitivity and specificity associated with various combinations above-mentioned, an ROC curve was plotted with “100% - specificity” on horizontal axis and “sensitivity” on vertical axis. And the AUROC was subsequently calculated. The AUROC is an indicator of the discriminatory ability, which is independent of diagnostic threshold. The value of AUROC ranges from 0.5 (no discriminatory power) to 1.0 (perfect discrimination). The AUROC for the PSC new guidelines and the AUROC for the old classification were compared by the method of DeLong in order to testify the advantage of the new classification system over the old one.

## SUPPLEMENTARY MATERIALS FIGURES AND TABLES


