# Supplementary material for: Papanicolaou Society of Cytopathology new guidelines have a greater ability of risk stratification for pancreatic endoscopic ultrasound-guided fine-needle aspiration specimens
Source: Oncotarget. 2016 Dec 22;8(5):8154–61. doi: 10.18632/oncotarget.14105 (PMC5352390; doi:10.18632/oncotarget.14105)
Supplement: Supplementary file 1 [file oncotarget-08-8154-s001.pdf]

Papanicolaou Society of Cytopathology new guidelines have a greater ability of risk stratification for pancreatic endoscopic ultrasound-guided fine-needle aspiration specimens

SUPPLEMENTARY TABLES

Supplementary Table 1: The Specified Histologic Subtypes Correlating to Each PSC Categories

PSC categories	Histological and Clinical Follow-up	Number of Cases	Final Diagnoses Subgroup
Nondiagnostic	NEM	5	NN
	SPN	2	N w/o HGM
	PanNET	2	N w/o HGM
	PDAC	12	N w/t HGM
Negative	NEM	65	NN
	SPN	2	N w/o HGM
	PanNET	1	N w/o HGM
	PDAC	15	N w/t HGM
Atypical	NEM	3	NN
	SPN	1	N w/o HGM
	PDAC	7	N w/t HGM
	SmCC	1	N w/t HGM
	acinar cell carcinoma	1	N w/t HGM
Neoplastic	SPN	4	N w/o HGM
	PanNET	9	N w/o HGM
	IPMN	2	N w/o HGM
	SmCC	1	N w/t HGM
	IPMN with associcated invasive adenocarcinoma	2	N w/t HGM
	PanNET mixed with mucinous adenocairnoma	2	N w/t HGM
Suspicious	NEM	1	NN
	PanNET	2	N w/o HGM
	IPMN	1	N w/o HGM
	PDAC	26	N w/t HGM
	SmCC	1	N w/t HGM
	non-Hodgkin lymphoma	1	N w/t HGM
Positive	PDAC	113	N w/t HGM
	SmCC	3	N w/t HGM
	undifferentiated carcinoma	2	N w/t HGM
	adenosquamous carcinoma	2	N w/t HGM
	metastatic spindle cell sarcoma	1	N w/t HGM
	MANEC	1	N w/t HGM
	non-Hodgkin lymphoma	2	N w/t HGM
	acinar cell carcinoma	1	N w/t HGM

Abbreviation: NEM, no (clinical, radiological and histologic) evidence of malignancy; PDAC, pancreatic ductal adenocarcinoma; SPN, solid pseudopapillary neoplasm; PanNET, pancreatic neuroendocrine tumor G1 or G2; IPMN, intraductal papillary mucinous neoplasm; SmCC, small cell carcinoma; MANEC, mixed adenoneuroendocrine carcinoma; NN, Non-Neoplastic; N w/o HGM, Neoplastic Without High Grade Malignancy; N w/t HGM, Neoplastic With High Grade Malignancy.

Supplementary Table 2: Absolute Risk and Relative Risk of Malignancy for Each Conventionally Old Categories

Cytologic Category	Absolute Risk (95% CI)	Relative Risk (95% CI)	*P* (Relative to Negative)	*P* (Relative to Positive)
"Old" Negative	26.0 (17.5-34.4)	1.00	-	<0.0001
"Old" Atypical	69.2 (44.1-94.3)	2.67 (1.64-4.34)	<0.0001	0.1803
"Old" Suspicious	87.5 (76.0-99.0)	3.37 (2.38-4.78)	<0.0001	0.8199
"Old" Positive	89.0 (83.9-94.1)	3.43 (2.46-4.76)	<0.0001	-

Supplementary Table 3: Sensitivity, Specificity and Accuracy Rate Associated with Various Combinations of Old Cytologic Categories

Cut-Point	Sensitivity	Specificity	Accuracy Rate	Youden Index
≥“Old” Negative	100.00%	0.00%	65.65%	0.0000
≥“Old” Atypical	86.01%	76.24%	82.65%	0.6225
≥“Old” Suspicious	81.35%	80.20%	80.95%	0.6155
≥“Old” Positive	66.84%	84.16%	72.79%	0.5100

Cytologic categories were rearranged by an ascending order of absolute risk and were successively set as diagnostic threshold (cut-point).
